# The relationship between circulating vitamin D3 and subclinical atherosclerosis in an elderly Asian population

**DOI:** 10.1038/s41598-020-75391-0

**Published:** 2020-10-30

**Authors:** Ya-Wen Lu, Ruey-Hsing Chou, Li-Kuo Liu, Liang-Kung Chen, Po-Hsun Huang, Shing-Jong Lin

**Affiliations:** 1 Division of Cardiology, Department of Medicine, Taipei Veterans General Hospital, No. 201, Sec. 2, Shih-Pai Road, Taipei, Taiwan; 2 Department of Critical Care Medicine, Taipei Veterans General Hospital, Taipei, Taiwan; 3 Center for Geriatrics and Gerontology, Taipei Veterans General Hospital, No. 201, Sec. 2, Shih-Pai Road, Taipei, Taiwan; 4 Cardiovascular Research Center, National Yang-Ming University, Taipei, Taiwan; 5 Institute of Clinical Medicine, National Yang-Ming University, Taipei, Taiwan; 6 Aging and Health Research Center, National Yang-Ming University, Taipei, Taiwan; 7 Institute of Public Health, National Yang-Ming University, Taipei, Taiwan; 8 Taipei Medical University, Taipei, Taiwan; 9 Division of Cardiology, Heart Center, Cheng-Hsin General Hospital, Taipei, Taiwan

**Keywords:** Cardiology, Atherosclerosis, Nutrition

## Abstract

The current evidence regarding the association between vitamin D deficiency and cardiovascular diseases/metabolic disorders is contradictory and inconclusive. In this large-scale observational study, we investigated the relationship between the serum 25-hydroxy vitamin D3 [25(OH)D] concentration and subclinical atherosclerosis in an elderly Asian population. In the I-Lan longitudinal study (ILAS), 1798 elderly, aged 50 and older, were enrolled. For each subject, serum 25-hydroxy vitamin D3 [25(OH)D] concentration and demographic data were recorded. The participants were divided into two groups according to their serum 25(OH)D level (sufficient, > 20 ng/mL and deficient, ≤ 20 ng/mL). Carotid intima-media thickness (cIMT) was measured at bilateral common carotid arteries. Subclinical atherosclerosis was defined as a mean cIMT > 0.81 mm. The mean subject age was 64 ± 9 years old, and 604 (33.6%) were identified as having serum 25(OH)D level ≤ 20 ng/mL. Subjects with serum 25(OH)D level ≤ 20 ng/mL were younger, more likely to be female and smoker, and had a higher incidence of hypertension, dyslipidemia, and metabolic syndrome, compared to those with serum 25(OH)D level > 20 ng/mL. Additionally, patients with serum 25(OH)D level ≤ 20 ng/mL were associated with a lower risk of subclinical atherosclerosis (crude OR: 0.63, 95% CI 0.50–0.81, *p* < 0.001), according to univariate analysis. However, after adjusting for gender and age, serum 25(OH)D level ≤ 20 ng/mL was not a significant risk factor for subclinical atherosclerosis. Serum 25(OH)D level ≤ 20 ng/mL was not an independent risk factor for subclinical atherosclerosis in this large elderly Asian population. Association observed in the univariate analysis may be confounded by gender or comorbidities.

## Introduction

The way to get vitamin D was from diet, dietary supplements or sunlight and metabolized to 25-hydroxyvitamin D [25(OH)D] in liver. Serum level of 25(OH)D could be used to determine the vitamin D nutritional status^1,2^. Conventionally, 25(OH)D was transferred to active form, 1,25-dihydroxyvitamin D [1,25(OH)_2_D], in kidney for regulating the metabolism of calcium, phosphorus and bone^1,3^. Recent evidence suggests that vitamin D also has systemic nonskeletal effects that include modulation of cancer cell progression, the cardiovascular system, and multiple autoimmune diseases through the genomic and nongenomic (rapid-response) cellular signaling by regulation of vitamin D receptor (VDR), which is the binding site of the 1,25(OH)_2_D. VDR has been found in a variety of cells, including cardiomyocytes, endothelial cells, activated T cells, as well as breast, colon, and prostate cancer cells^3,4^. The active form of vitamin D—1,25(OH)_2_D may modulate the tissue matrix metalloproteinase (MMP) and anti-proteinase inhibitors (TIMP) system, which was found to be involved in atherosclerosis, whereas MMP level was decreased after vitamin D supplement^5,6^.

Current evidence regarding the association between vitamin D deficiency and atherosclerosis is contradictory and inconclusive^7–9^. Lowered vitamin D level has been reported to be one of risk factors for coronary heart disease in patients with type 2 diabetes mellitus^10^, chronic kidney disease^11^, and human immunodeficiency virus (HIV)-infected African Americans^12,13^ Vitamin D deficiency was also related to obesity, higher Framingham risk scores, waist circumference, and body mass index (BMI) in different ethnicities^14–16^. On the contrary, in a large longitudinal community-based cohort of 6459 participants, no association between vitamin D concentration and adverse cardiovascular events was found. Instead, vitamin D deficiency was reported to be associated with higher parathyroid hormone (PTH ≥ 65 pg/mL) level, increased left ventricular mass, and risk for incident heart failure^17^. Vitamin D supplementation has been inconsistently shown to reduce cardiovascular risk factors in a meta-analysis of randomized controlled studies, after adjusting blood pressure, lipid profile, insulin resistance, and vascular function^18–20^. Moreover, vitamin D supplementation has been suggested to decrease the risk of heart failure, but not that of myocardial infarction or stroke^21^.

Since current evidence regarding the association between vitamin D deficiency and cardiovascular disease/metabolic disorder is contradictory and inconclusive, in this large-scale observational study, we investigated the relationship between serum vitamin D concentration and subclinical atherosclerosis in elderly Asian population of the I-Lan Longitudinal Aging Study (ILAS), which included subjects aged 50 years and above without known active disease^22^. This is currently the largest study to investigate vitamin D deficiency and subclinical cardiovascular disease in an elderly Asian population.

## Results

A total of 1798 participants were analyzed and divided into two groups according to the serum 25(OH)D level (sufficient, > 20 ng/mL and deficient, ≤ 20 ng/mL). Mean age of the study population was 62.0 (55.9–71.1) years old, and 604 subjects (33.6%) were identified as having serum 25(OH)D ≤ 20 ng/mL. Subjects with serum 25(OH)D deficiency were more likely to be younger, female, and less smokers; and they had lower prevalence of hypertension, dyslipidemia, and lipid-lowering agent usage. In addition, they had higher estimated glomerular filtration rate (eGFR) and iPTH concentration as well as lower serum uric acid level, hip BMD, and cIMT. Baseline characteristics of the study cohort are summarized in Table 1.

**Table 1:** Baseline characteristics of patients grouped by serum 25(OH)D levels.

	Total N = 1798	Serum 25(OH)D ≥ 20 ng/mL, n = 1194	Serum 25(OH)D < 20 ng/mL, n = 604	*P* value
**Clinical profile**				
Age (years, median, (interquartile range))	62.0 (55.9–71.1)	64.6 (56.4–71.5)	61.0 (55.3–70.5)	0.005
Male, n (%)	855 (48.0)	688 (57.6)	167 (27.6)	< 0.001
Current Smoking, n (%)	331 (18.4)	255 (21.4)	76 (12.6)	< 0.001
Never smoking, n (%)	1250 (69.5)	765 (64.1)	485 (80.3)	
Current smokers, n (%)	331 (18.4)	255 (21.4)	76 (12.6)	
Quitted smoking, n (%)	217 (12.1)	174 (14.6)	43 (7.1)	
Hypertension, n (%)	740 (41.2)	512 (42.9)	228 (37.7)	0.038
Diabetes, n (%)	299 (16.6)	204 (17.1)	95 (15.7)	0.503
Dyslipidemia, n (%)	139 (7.7)	103 (8.7)	36 (5.9)	0.018
Metabolic syndrome, n (%)	610 (33.9)	374 (31.3)	236 (39.1)	0.001
Waist (cm)	84.5 (78.0–91.0)	85.0 (78.5–91.0)	84.0 (77.0–91.0)	0.282
BMI (kg/m^2^)	24.54 (22.29–26.85)	24.36 (22.27–26.60)	24.92 (22.31–27.37)	0.006
Antihypertensive agents, n (%)	386 (21.5)	268 (22.7)	118 (19.2)	0.050
Lipid-lowering agents, n (%)	127 (7.1)	94 (7.9)	33 (5.4)	0.025
**Biochemical & imaging studies median, (interquartile range)**				
eGFR (mL/min)	77.39 (60.75–98.66)	73.74 (57.67–93.21)	85.88 (67.97–106.04)	< 0.001
FBG	96.0 (89.0–105.0)	96.0 (89.0–105.0)	95.0 (89.0–106.0)	0.597
HOMA-IR	1.79 (1.08–2.93)	1.64 (1.02–2.76)	2.14 (1.22–3.21)	< 0.001
LDL-c (mg/dL)	117.0 (96.0–139.0)	116.0 (95.0–137.3)	120.0 (98.0–142.0)	0.050
HDL	52.5 (45.0–62.0)	52.0 (45.0–61.0)	54.0 (46.0–65.0)	0.004
TG	105.0 (76.0–145.0)	98.0 (73.0–138.0)	116.0 (84.0–159.8)	< 0.001
Uric acid (mg/dL)	5.7 (4.8–6.8)	5.9 (5.0–6.9)	5.4 (4.6–6.5)	< 0.001
hsCRP	0.090 (0.038–0.224)	0.089 (0.037–0.229)	0.091 (0.040–0.220)	0.667
iPTH	39.10 (29.80–50.40)	36.75 (28.58–47.33)	44.10 (32.90–56.50)	< 0.001
VitD3	22.5 (18.7–27.1)	25.4 (22.6–29.7)	17.1 (14.8–18.7)	< 0.001
hip BMD	0.83 (0.74–0.93)	0.84 (0.75–0.94)	0.82 (0.72–0.91)	< 0.001
Total body fat % (DXA)	32.10 (25.30–37.98)	29.95 (23.60–36.20)	35.85 (29.40–40.40)	< 0.001
cIMT	0.70 (0.60–0.75)	0.70 (0.60–0.80)	0.70 (0.60–0.75)	0.008

Values are presented as the median with interquartile range or n (%).

*BMI* body mass index, *eGFR* estimated glomerular filtration rate, *FBG* fasting blood glucose, *HOMA-IR* homeostatic model assessment, *LDL-c* low-density lipoprotein cholesterol, *HDL* high-density lipoprotein, *TG* triglyceride, *hsCRP* high-sensitivity C-reactive protein, *iPTH* intact parathyroid hormone, *VitD3* vitamin D3, *BMD* bone mineral density, *DXA* dual-energy X-ray absorptiometry, *cIMT* carotid intima-media thickness.

Table 2 lists the correlation coefficients between cIMT and serum 25(OH)D concentration with other variables. Age, male gender, current smoker, hypertension, antihypertensive drug usage, and serum uric acid level were positively correlated with both cIMT and serum 25(OH)D. Meanwhile, serum HDL level, eGFR, and total body fat were negatively correlated with both cIMT and serum 25(OH)D concentration. Metabolic syndrome, BMI, FBG, and HOMA-IR were negatively related to serum 25(OH)D concentration but positively associated with cIMT. Additionally, serum LDL, TG, and iPTH levels were negatively correlated with serum 25(OH)D concentration but were not associated with cIMT (p < 0.01).

**Table 2:** Correlation coefficients of the serum 25(OH)D concentration and the carotid intima-media thickness with other cardiovascular risk factors.

	Serum 25(OH)D	cIMT
**Clinical profiles**		
Age	0.110**	0.425**
Male gender	0.361**	0.219**
Smoking	0.142**	0.118**
Hypertension	0.047*	0.202**
Diabetes	0.025	0.104**
Dyslipidemia	0.046	0.018
Metabolic syndrome	− 0.090**	0.105**
Waist	0.035	0.196**
BMI	− 0.096**	0.066**
Antihypertensive agents	0.047*	0.124**
Lipid-lowering agents	0.043	0.031
**Biochemical and image studies**		
eGFR	− 0.254**	− 0.315**
FBG	− 0.053*	0.057*
HOMA-IR	− 0.115**	0.052*
LDL	− 0.091**	− 0.026
HDL	− 0.082**	− 0.149**
TG	− 0.152**	0.035
Uric acid	0.179**	0.162**
hsCRP	0.018	0.116
iPTH	− 0.118**	− 0.004
VitD3	–	0.105**
Hip BMD	0.132**	− 0.021
Total body fat (%) (DXA)	− 0.341**	− 0.117**
cIMT	0.105**	–

*BMI* body mass index, *eGFR* estimated glomerular filtration rate, *FBG* fasting blood glucose, *HOMA-IR* homeostatic model assessment, *LDL* low-density lipoprotein, *HDL* high-density lipoprotein, *TG* triglyceride, *hsCRP* high-sensitivity C-reactive protein, *iPTH* intact parathyroid hormone, *VitD3* vitamin D3, *BMD* bone mineral density, *DXA* dual-energy X-ray absorptiometry, *cIMT* carotid intima-media thickness.

*Correlation is significant at the 0.05 level (2-tailed).

**Correlation is significant at the 0.01 level (2 tailed).

Compared to subjects with serum 25(OH)D sufficiency, patients with serum 25 (OH)D deficiency were associated with lower risk of subclinical atherosclerosis (crude OR: 0.63, 95% CI 0.50–0.81, *p* < 0.001), according to univariate analysis (Table 3, model 1). After adjusting for age, gender, smoking, BMI, diabetes mellitus, serum 25(OH)D < 20 ng/mL was not associated with increased risk of subclinical atherosclerosis (adjusted OR: 0.84, 95% CI 0.61–1.14, *p* = 0.256) (Table 3, model 2), and similar findings were observed after adjusting variables that significantly affect serum 25(OH)D concentration including age, gender, smoking, BMI, diabetes mellitus and eGFR (adjusted OR: 0.93, 95% CI 0.69–1.24, *p* = 0.599) (Table 3, model 3). Subgroup analyses of the association between serum 25(OH)D < 20 ng/mL and subclinical atherosclerosis are presented in Table 4. In multivariate regression model, the results showed no significant association between 25(OH)D < 20 ng/mL and subclinical atherosclerosis in different subgroups, including age ≥ 65 years old, male gender, current smoker, other co-morbidities such as hypertension, diabetes, dyslipidemia, metabolic syndrome, and higher BMI.

**Table 3:** Univariate and multivariate logistic regression analysis for vitamin D3 (VitD3) deficiency and the risk of subclinical atherosclerosis (defined as carotid intima-media thickness, cIMT > 0.8 mm).

Subclinical arthrosclerosis (cIMT > 0.8 mm)	Odds ratio	95% Confidence Interval	*P* value
**Model 1**			
25(OH)D sufficiency (> 20)	Reference	–	–
25(OH)D deficiency (≤ 20)	0.63	0.50–0.81	< 0.001
**Model 2**			
25(OH)D sufficiency (> 20)	Reference	–	–
25(OH)D deficiency (≤ 20)	0.84	0.61–1.14	0.256
**Model 3**			
25(OH)D sufficiency (> 20)	Reference	–	–
25(OH)D deficiency (≤ 20)	0.93	0.69–1.24	0.599

*Model 1* adjusted for VitD3 (univariate), *Model 2* adjusted for age, gender, smoking, BMI, diabetes mellitus, *Model 3* adjusted for age, gender, smoking, BMI, diabetes mellitus, eGFR.

**Table 4:** Subgroup analysis of the association of serum 25(OH)D deficiency and subclinical arthrosclerosis (carotid intima-media thickness, cIMT > 0.8 mm).

Subgroup (n)	Crude OR (95% CI)	*P* value	Adjusted OR (95% CI)*	*P* value*
Overall (n = 1798)	0.63 (0.50–0.81)	< 0.001	0.93 (0.69–1.24)	0.599
**Age**				
≥ 65 (n = 734)	0.68 (0.49–0.94)	0.021	1.02 (0.70–1.49)	0.936
< 65 (n = 1064)	0.62 (0.42–0.93)	0.021	0.79 (0.50–1.26)	0.325
**Gender**				
Male (n = 855)	0.95 (0.66–1.37)	0.775	1.17 (0.76–1.81)	0.487
Female (n = 943)	0.69 (0.48–0.98)	0.038	0.75 (0.50–1.13)	0.169
**Current smoker**				
Yes (n = 331)	0.60 (0.33–1.08)	0.088	0.75 (0.35–1.62)	0.461
No (n = 1467)	0.674 (0.52–0.88)	0.004	0.93 (0.68–1.29)	0.676
**Hypertension**				
Yes (n = 740)	0.73 (0.52–1.03)	0.073	0.99 (0.65–1.52)	0.978
No (n = 1058)	0.58 (0.41–0.83)	0.003	0.82 (0.54–1.26)	0.374
**Diabetes**				
Yes (n = 299)	0.60 (0.35–1.05)	0.076	0.75 (0.38–1.50)	0.418
No (n = 1499)	0.64 (0.49–0.88)	0.001	0.96 (0.69–1.33)	0.798
**Dyslipidemia**				
Yes (n = 139)	0.59 (0.23–1.49)	0.261	0.93 (0.25–3.56)	0.920
No (n = 1659)	0.64 (0.50–0.82)	< 0.001	0.96 (0.71–1.30)	0.787
**Metabolic syndrome**				
Yes (n = 610)	0.613 (0.42–0.90)	0.012	0.83 (0.52–1.32)	0.421
No (n = 1188)	0.62 (0.45–0.85)	0.003	1.05 (0.71–1.56)	0.796
**BMI**				
< 24.9 (n = 993)	0.52 (0.36–0.75)	0.001	0.79 (0.50–1.24)	0.298
25.0–29.9 (n = 666)	0.72 (0.50–1.04)	0.077	1.01 (0.65–1.56)	0.974
≥ 30 (n = 139)	0.70 (0.32–1.53)	0.371	1.02 (0.28–3.73)	0.974

*Adjusted for age, gender, BMI, eGFR, smoking, hypertension, diabetes mellitus, metabolic syndrome, eGFR, antihypertensive agent usage, waist circumference, FBG, HOMA-IR, HDL, LDL, TG, UA, iPTH, hip BMD, and total body fat (%, DXA).

## Discussion

### Main finding

In this large observational cohort study composed of 1798 Asians older than 50 years , prevalence of vitamin D deficiency was 33.6% (n = 604). After adjusting the traditional variables associated with vitamin D deficiency and further subgroup analyses, the results showed that vitamin D deficiency was not an independent risk factor for subclinical atherosclerosis. Significant association observed in the univariate analysis may be confounded by gender and age difference or comorbidities.

### Current evidence of vitamin D deficiency and atherosclerosis

Vitamin D deficiency and cIMT have been studied in healthy populations and various specific groups, but inconsistent results were found, even within populations. In a prior study composed of patients with type 1 diabetes, deficiency of circulating 25(OH)D (< 20 ng/mL) was associated with a 3.3-fold increased odds ratio of arterial calcification than serum 25(OH)D sufficient group (≥ 30 ng/mL) after adjusting age, gender, and hours of daylight exposure^23^. However, contrary outcome was found in another cohort study of 1193 subjects with type 1 diabetes mellitus, which suggested that lower serum 25(OH)D level was related to lower mean coronary arterial calcium^24^. In those with type 1 diabetes mellitus with mean age around 30–35 years old, previous studies have reported no significant association between the circulating serum 25(OH)D concentration and cIMT^24,25^. In a cross-sectional cohort study of 654 relatively healthy participants without history of coronary heart disease, serum 25(OH)D concentration was significantly inversely related to the internal cIMT but not the common cIMT^26^ and similar finding was found in another study composed of HIV-infected patients^27^.

Inconsistent results were also found in different meta-analysis. The enrolled studies in meta-analysis had significant heterogeneity although vitamin D deficiency (serum 25(OH)D < 20 ng/mL) was found to be associated with a higher cIMT; besides, in female patients with a higher BMI, waist circumference, or eGFR, vitamin D deficiency was associated with lower cIMT^28,29^. These findings indicate that the relationship between vitamin D deficiency and subclinical atherosclerosis is not consistent. In the present study, the association of serum 25(OH)D and subclinical atherosclerosis could vary by age, gender, and underlying diseases. After the covariates were adjusted, serum 25(OH)D deficiency was not an independent risk factor of subclinical atherosclerosis although positive correlation between age and serum 25(OH)D existed.

### Relationship between serum 25(OH)D and atherosclerosis: contributor or bystander?

Active form of Vitamin D—[1,25(OH)D] may influence arterial stiffness directly via regulation of nuclear vitamin D receptor (VDR) or indirect pathway that 1,25(OH)D may relate to blood pressure controlled by suppression of Renin–Angiotensin–Aldosterone System (RAAS), in VDR-knockout mice^30,31^. The relationship between vitamin D deficiency and subclinical atherosclerosis may be confounded by patient’s age and physical characteristics. A nationwide study in Taiwan revealed that subjects with higher vitamin D level were younger; had lower education status, BMI, and vegetable intake; and higher level of work-related physical activity and fish intake^32^. The vitamin D deficiency group in our cohort was significantly younger, lesser comorbidities with hypertension, diabetes, dyslipidemia, antihypertensive drug usage, and lipid-lowering agent usage; but it had higher prevalence of subjects with metabolic syndrome, and increase in BMI, HOMA-IR and total body fat. Thus, association between vitamin D deficiency and cIMT became insignificant after adjusting age and gender in our study.

### Study limitations

There are several limitations of the present study that must be acknowledged. First, due to the cross-sectional study design, causal relationship between vitamin D and atherosclerosis could not be fully assessed. In addition, some confounding factors, such as duration of sun exposure, vitamin D supplementation, betel-quid chewing were not taken into account in the present study. Third, this is a cross-section cohort study and was lack of CV outcomes of long-term follow-up like longitudinal study; thus, we could not investigate the impact of insufficient serum 25(OH)D level on risk of CVD.

## Conclusions

Prevalence of vitamin D deficiency (defined by serum level of 25(OH)D) was approximately one-third in the elderly Asian population in this study. Our findings suggested that vitamin D deficiency is not an independent predictor of subclinical atherosclerosis in elderly Asians after adjusting confounding factors. Future studies to address the association between serum 25(OH)D and the cIMT need to be explored carefully.

## Methods

### Study population

The present study used the sample data from the I-Lan Longitudinal Aging Study (ILAS), which is a research cohort of community-dwelling adults aged more than 50 years old who were randomly recruited through household registration records^33^. The survey enrolled 1839 community-dwelling older adults from August 2011 to August 2013. The participants were excluded from this study if any of the following conditions were met: (1) unable to cooperate or communicate with study investigators; (2) declined to grant consent; (3) currently institutionalized^34^; (4) had any known active disease, such as active cancer, sepsis, heart failure, chronic obstructive pulmonary disease, or functional dependence; (5) had a life-expectancy of less than 6 months; and (6) planned to leave I-Lan county. This study was conducted by Institutional Review Board of National Yang-Ming University approval. All participants provided written informed consent. All methods were performed in accordance with the relevant guidelines and regulations.

### Anthropometry and demographic measurements

The demographic and medical details of the participants were collected by the research nurse, who also performed the anthropometric measurements of the patients, including weight, height, BMI, and waist circumference. Brachial blood pressure was measured with a mercury sphygmomanometer after the subjects had rested for at least 15 min. Basic medical histories, including underlying diseases, medication, and whether or not the current smokers (including no smoking, current smoker or quitted smoking), were obtained from personal interviews and recorded medical notes. Metabolic syndrome was defined according to the criteria proposed by Taiwan’s Ministry of Health and Welfare, with more than three of the following risk determinants: (1) waist circumference > 90 cm for men or > 80 cm for women; (2) systolic blood pressure ≥ 130 mmHg, diastolic blood pressure ≥ 85 mmHg, or taking antihypertensive agents; (3) high-density lipoprotein (HDL) < 40 mg/dL for men or < 50 mg/dL for women^34^; (4) triglyceride (TG) ≥ 150 mg/dL; (5) fasting blood glucose (FBG) ≥ 100 mg/dL or taking antihyperglycemic agents^35,36^. The body composition, including the total fat mass and the bone mineral density (BMD), was measured by whole-body dual-energy X-ray absorptiometry. The total body fat percentage was calculated as the total fat mass divided by the total body mass times 100^37^.

### Laboratory examinations

After fasting for at least 10 h, peripheral blood samples were collected at 7–9 AM to determine the concentrations of hemoglobin A1c, FBG, total cholesterol, HDL, low-density lipoprotein (LDL), TG, uric acid, and high-sensitivity C-reactive protein (hsCRP) by using an automatic analyzer (ADVIA 1800; Siemens, Malvern, PA, USA). Insulin resistance was quantified with the homeostatic model assessment (HOMA-IR) using the following formula: HOMA-IR = glucose (mmol/L) × insulin (μU/mL)/22.5^38^.

Serum concentration of 25(OH)D were determined by using the commercially available LIAISON 25 OH vitamin D total assay from DiaSorin (DiaSorin vit D; DiaSorin Inc., Northwestern Avenue, MN, USA). The assay range was 4.0–150 ng/mL. The intra- and interassay coefficients were 2.9–5.5% and 6.3–7.9%, respectively. Intact parathyroid hormone (iPTH) were measured by chemiluminescence immunoassays (ADVIA Centaur, Siemens, USA)^39^. According to the recommendation of the US Preventive Services Task Force, vitamin D deficiency was defined as a VitD3 concentration less than 20 ng/mL^40^.

### Assessment of carotid intima-media thickness (cIMT) and subclinical atherosclerosis

The cIMT was measured using a high-resolution, broad-width, linear array transducer (LOGIQ 400 PRO; GE, Cleveland, OH, USA) at the level of the common carotid artery. All examinations were carried out by the same trained technician, who measured the arteries including the proximal to distal parts of the bilateral common carotid arteries on longitudinal views^33^. The mean cIMT was defined as the average of the right-side and left-side cIMT values. Subclinical atherosclerosis was defined as a mean cIMT > 0.81 mm; this value has been demonstrated to be associated with an increased risk of stroke and coronary events^41,42^.

### Statistical analysis

All continuous descriptive variables are expressed as the median with interquartile range due to the non-normal distribution, and categorical variables are expressed as numbers (percentages). Comparison between two groups was analyzed by the Mann Whitney u test for continuous variables, and Fisher’s exact test for categorical variables. Pearson’s correlation test was used to assess the correlations between serum 25(OH)D and cIMT with other variables. Logistic regression analysis was performed to assess the relationships between the 25(OH)D concentration and subclinical atherosclerosis. Variables significantly correlated to the cIMT were further entered into multivariate regression analysis. To investigate the association of 25(OH)D on subclinical atherosclerosis modified by different comorbidities, we performed subgroup analyses stratified by age, gender, smoking status, hypertension, diabetes, dyslipidemia, metabolic syndrome, and BMI. Statistical analyses were performed using SPSS version 22.0 (SPSS Inc., Chicago, IL, USA). *P* values < 0.05 were considered to be statistically significant.

## Data Availability

The datasets generated and analyzed in the current study are available from the corresponding author on reasonable request.

## References

[1] Howe WR, Dellavalle R (2007). Vitamin D deficiency. N. Engl. J. Med..

[2] Khundmiri SJ, Murray RD, Lederer E (2016). PTH and vitamin D. Comprehens. Physiol..

[3] Christakos S, Dhawan P, Verstuyf A, Verlinden L, Carmeliet G (2016). Vitamin D: Metabolism, molecular mechanism of action, and pleiotropic effects. Physiol. Rev..

[4] Mizwicki MT, Norman AW (2009). The vitamin D sterol-vitamin D receptor ensemble model offers unique insights into both genomic and rapid-response signaling. Sci. Signal..

[5] Qin X, Corriere MA, Matrisian LM, Guzman RJ (2006). Matrix metalloproteinase inhibition attenuates aortic calcification. Arterioscler. Thromb. Vasc. Biol..

[6] Timms PM, Mannan N, Hitman GA, Noonan K, Mills PG, Syndercombe-Court D, Aganna E, Price CP, Boucher BJ (2002). Circulating MMP9, vitamin D and variation in the TIMP-1 response with VDR genotype: mechanisms for inflammatory damage in chronic disorders?. QJM Month. J. Assoc. Physicians..

[7] Kassi E, Adamopoulos C, Basdra EK, Papavassiliou AG (2013). Role of vitamin D in atherosclerosis. Circulation.

[8] Wang TJ, Pencina MJ, Booth SL, Jacques PF, Ingelsson E, Lanier K, Benjamin EJ, D'Agostino RB, Wolf M, Vasan RS (2008). Vitamin D deficiency and risk of cardiovascular disease. Circulation.

[9] Ford ES, Ajani UA, McGuire LC, Liu S (2005). Concentrations of serum vitamin D and the metabolic syndrome among U.S. adults. Diabetes Care.

[10] Heidari B, Nargesi AA, Hafezi-Nejad N, Sheikhbahaei S, Pajouhi A, Nakhjavani M, Esteghamati A (2015). Assessment of serum 25-hydroxy vitamin D improves coronary heart disease risk stratification in patients with type 2 diabetes. Am. Heart J..

[11] Gluba-Brzózka A, Franczyk B, Ciałkowska-Rysz A, Olszewski R, Rysz J (2018). Impact of vitamin D on the cardiovascular system in advanced chronic kidney disease (CKD) and dialysis patients. Nutrients..

[12] Lai H, Fishman EK, Gerstenblith G, Moore R, Brinker JA, Keruly JC, Chen S, Detrick B, Lai S (2013). Vitamin D deficiency is associated with development of subclinical coronary artery disease in HIV-infected African American cocaine users with low Framingham-defined cardiovascular risk. Vasc. Health Risk Manag..

[13] Lai H, Fishman EK, Gerstenblith G, Brinker JA, Tong W, Bhatia S, Detrick B, Lai S (2012). Vitamin D deficiency is associated with significant coronary stenoses in asymptomatic African American chronic cocaine users. Int. J. Cardiol..

[14] Chiang JM, Stanczyk FZ, Kanaya AM (2018). Vitamin D levels, body composition, and metabolic factors in Asian Indians: Results from the metabolic syndrome and atherosclerosis in South Asians living in America pilot study. Ann. Nutr. Metab..

[15] Zhu, W. & Heil, D.P. Associations of vitamin D status with markers of metabolic health: A community-based study in Shanghai, China. *Diabet. Metab. Syndr*. 2018. 10.1016/j.dsx.2018.04.010 29699952

[16] Al-Khalidi B, Kimball SM, Kuk JL and Ardern CI. Metabolically healthy obesity, vitamin D, and all-cause and cardiometabolic mortality risk in NHANES III. *Clin. Nutr. (Edinburgh, Scotland)*. 2018. 10.1016/j.clnu.2018.02.025 29525513

[17] Bansal N, Zelnick L, Robinson-Cohen C, Hoofnagle AN, Ix JH, Lima JA, Shoben AB, Peralta CA, Siscovick DS, Kestenbaum B, de Boer IH (2014). Serum parathyroid hormone and 25-hydroxyvitamin D concentrations and risk of incident heart failure: the Multi-Ethnic Study of Atherosclerosis. J. Am. Heart Assoc..

[18] Kubiak J, Thorsby PM, Kamycheva E, Jorde R (2018). Vitamin D supplementation does not improve CVD risk factors in vitamin D-insufficient subjects. Endocr. Connect..

[19] Beveridge LA, Khan F, Struthers AD, Armitage J, Barchetta I, Bressendorff I, Cavallo MG, Clarke R, Dalan R, Dreyer G, Gepner AD, Forouhi NG, Harris RA, Hitman GA, Larsen T, Khadgawat R, Marckmann P, Mose FH, Pilz S, Scholze A, Shargorodsky M, Sokol SI, Stricker H, Zoccali C, Witham MD (2018). Effect of vitamin D supplementation on markers of vascular function: A systematic review and individual participant meta-analysis. J. Am. Heart Assoc..

[20] Swart KM, Lips P, Brouwer IA, Jorde R, Heymans MW, Grimnes G, Grubler MR, Gaksch M, Tomaschitz A, Pilz S, Eiriksdottir G, Gudnason V, Wamberg L, Rejnmark L, Sempos CT, Durazo-Arvizu RA, Dowling KG, Hull G, Skrabakova Z, Kiely M, Cashman KD, van Schoor NM (2018). Effects of vitamin D supplementation on markers for cardiovascular disease and type 2 diabetes: An individual participant data meta-analysis of randomized controlled trials. Am. J. Clin. Nutr..

[21] Ford JA, MacLennan GS, Avenell A, Bolland M, Grey A, Witham M, for the RTG (2014). Cardiovascular disease and vitamin D supplementation: trial analysis, systematic review, and meta-analysis. Am. J. Clin. Nutr..

[22] Chen CH, Liu LK, Chen MJ, Lee WJ, Lin MH, Peng LN, Chen LK (2018). Associations between vitamin D deficiency, musculoskeletal health, and cardiometabolic risk among community-living people in Taiwan: Age and sex-specific relationship. Medicine..

[23] Young KA, Snell-Bergeon JK, Naik RG, Hokanson JE, Tarullo D, Gottlieb PA, Garg SK, Rewers M (2011). Vitamin D deficiency and coronary artery calcification in subjects with type 1 diabetes. Diabetes Care.

[24] Sachs MC, Brunzell JD, Cleary PA, Hoofnagle AN, Lachin JM, Molitch ME, Steffes MW, Zinman B, de Boer IH (2013). Circulating vitamin D metabolites and subclinical atherosclerosis in type 1 diabetes. Diabetes Care.

[25] Serra-Planas E, Aguilera E, Granada ML, Soldevila B, Salinas I, Reverter JL, Pizarro E, Pellitero S, Alonso N, Mauricio D, Puig-Domingo M (2015). High prevalence of vitamin D deficiency and lack of association with subclinical atherosclerosis in asymptomatic patients with Type 1 Diabetes Mellitus from a Mediterranean area. Acta Diabetol..

[26] Reis JP, von Muhlen D, Michos ED, Miller ER, Appel LJ, Araneta MR, Barrett-Connor E (2009). Serum vitamin D, parathyroid hormone levels, and carotid atherosclerosis. Atherosclerosis..

[27] Huff H, Merchant AT, Lonn E, Pullenayegum E, Smaill F, Smieja M (2018). Vitamin D and progression of carotid intima-media thickness in HIV-positive Canadians. HIV Med..

[28] Lupoli R, Vaccaro A, Ambrosino P, Poggio P, Amato M, Di Minno MN (2017). Impact of Vitamin D deficiency on subclinical carotid atherosclerosis: A pooled analysis of cohort studies. J. Clin. Endocrinol. Metab..

[29] Targher G, Bertolini L, Padovani R, Zenari L, Scala L, Cigolini M, Arcaro G (2006). Serum 25-hydroxyvitamin D3 concentrations and carotid artery intima-media thickness among type 2 diabetic patients. Clin. Endocrinol..

[30] Al Mheid I, Quyyumi AA (2017). Vitamin D and cardiovascular disease: Controversy unresolved. J. Am. Coll. Cardiol..

[31] Li YC, Kong J, Wei M, Chen ZF, Liu SQ, Cao LP (2002). 1,25-Dihydroxyvitamin D(3) is a negative endocrine regulator of the renin-angiotensin system. J. Clin. Investig..

[32] Chuang SC, Chen HL, Tseng WT, Wu IC, Hsu CC, Chang HY, Chen YI, Lee MM, Liu K, Hsiung CA (2016). Circulating 25-hydroxyvitamin D and physical performance in older adults: A nationwide study in Taiwan. Am. J. Clin. Nutr..

[33] Liu LK, Lee WJ, Chen LY, Hwang AC, Lin MH, Peng LN, Chen LK (2014). Sarcopenia, and its association with cardiometabolic and functional characteristics in Taiwan: Results from I-Lan Longitudinal Aging Study. Geriatr. Gerontol. Int..

[34] Vimaleswaran KS, Cavadino A, Berry DJ, Jorde R, Dieffenbach AK, Lu C, Alves AC, Heerspink HJ, Tikkanen E, Eriksson J, Wong A, Mangino M, Jablonski KA, Nolte IM, Houston DK, Ahluwalia TS, van der Most PJ, Pasko D, Zgaga L, Thiering E, Vitart V, Fraser RM, Huffman JE, de Boer RA, Schottker B, Saum KU, McCarthy MI, Dupuis J, Herzig KH, Sebert S, Pouta A, Laitinen J, Kleber ME, Navis G, Lorentzon M, Jameson K, Arden N, Cooper JA, Acharya J, Hardy R, Raitakari O, Ripatti S, Billings LK, Lahti J, Osmond C, Penninx BW, Rejnmark L, Lohman KK, Paternoster L, Stolk RP, Hernandez DG, Byberg L, Hagstrom E, Melhus H, Ingelsson E, Mellstrom D, Ljunggren O, Tzoulaki I, McLachlan S, Theodoratou E, Tiesler CM, Jula A, Navarro P, Wright AF, Polasek O, Wilson JF, Rudan I, Salomaa V, Heinrich J, Campbell H, Price JF, Karlsson M, Lind L, Michaelsson K, Bandinelli S, Frayling TM, Hartman CA, Sorensen TI, Kritchevsky SB, Langdahl BL, Eriksson JG, Florez JC, Spector TD, Lehtimaki T, Kuh D, Humphries SE, Cooper C, Ohlsson C, Marz W, de Borst MH, Kumari M, Kivimaki M, Wang TJ, Power C, Brenner H, Grimnes G, van der Harst P, Snieder H, Hingorani AD, Pilz S, Whittaker JC, Jarvelin MR, Hypponen E (2014). Association of vitamin D status with arterial blood pressure and hypertension risk: A mendelian randomisation study. Lancet Diabet. Endocrinol..

[35] Alexander CM, Landsman PB, Teutsch SM, Haffner SM (2003). NCEP-defined metabolic syndrome, diabetes, and prevalence of coronary heart disease among NHANES III participants age 50 years and older. Diabetes.

[36] Hwang LC, Bai CH, Chen CJ (2006). Prevalence of obesity and metabolic syndrome in Taiwan. J. Formosan Med. Assoc..

[37] Peng LN, Lee WJ, Liu LK, Lin MH, Chen LK (2018). Healthy community-living older men differ from women in associations between myostatin levels and skeletal muscle mass. J. Cachexia Sarcopenia Muscle.

[38] Wallace TM, Levy JC, Matthews DR (2004). Use and abuse of HOMA modeling. Diabetes Care.

[39] Liu LK, Lee WJ, Chen LY, Hwang AC, Lin MH, Peng LN, Chen LK (2015). Association between frailty, osteoporosis, falls and hip fractures among community-dwelling people aged 50 years and older in Taiwan: Results from I-lan longitudinal aging study. PLoS ONE.

[40] LeFevre ML (2015). Screening for vitamin D deficiency in adults: U.S. preventive services task force recommendation statement. Ann. Intern. Med..

[41] Rosvall M, Janzon L, Berglund G, Engstrom G, Hedblad B (2005). Incidence of stroke is related to carotid IMT even in the absence of plaque. Atherosclerosis..

[42] Rosvall M, Janzon L, Berglund G, Engstrom G, Hedblad B (2005). Incident coronary events and case fatality in relation to common carotid intima-media thickness. J. Intern. Med..

